# Randomized Clinical Trial of Thrice-Weekly 4-Month Moxifloxacin or Gatifloxacin Containing Regimens in the Treatment of New Sputum Positive Pulmonary Tuberculosis Patients

**DOI:** 10.1371/journal.pone.0067030

**Published:** 2013-07-03

**Authors:** Mohideen S. Jawahar, Vaithilingam V. Banurekha, Chinnampedu N. Paramasivan, Fathima Rahman, Rajeswari Ramachandran, Perumal Venkatesan, Rani Balasubramanian, Nagamiah Selvakumar, Chinnaiyan Ponnuraja, Allaudeen S. Iliayas, Navaneethapandian P. Gangadevi, Balambal Raman, Dhanaraj Baskaran, Santhanakrishnan R. Kumar, Marimuthu M. Kumar, Victor Mohan, Sudha Ganapathy, Vanaja Kumar, Geetha Shanmugam, Niruparani Charles, Murugesan R. Sakthivel, Kannivelu Jagannath, Chockalingam Chandrasekar, Ramavaram T. Parthasarathy, Paranji R. Narayanan

**Affiliations:** 1 National Institute for Research in Tuberculosis (formerly Tuberculosis Research Centre), Chennai, India; 2 National Institute for Research in Tuberculosis (formerly Tuberculosis Research Centre), Madurai, India; 3 Institute of Thoracic Medicine, Chennai, India; 4 Government Rajaji Hospital, Madurai, India; 5 Government Tiruvatteeswarar Hospital, Chennai, India; Glaxo Smith Kline, Denmark

## Abstract

**Background:**

Shortening tuberculosis (TB) treatment duration is a research priority. This paper presents data from a prematurely terminated randomized clinical trial, of 4-month moxifloxacin or gatifloxacin regimens, in South India.

**Methods:**

Newly diagnosed, sputum-positive HIV-negative pulmonary TB patients were randomly allocated to receive gatifloxacin or moxifloxacin, along with isoniazid and rifampicin for 4 months with pyrazinamide for first 2 months (G or M) or isoniazid and rifampicin for 6 months with ethambutol and pyrazinamide for first 2 months (C). All regimens were administered thrice-weekly. Clinical and bacteriological assessments were done monthly during treatment and for 24 months post-treatment. The Data and Safety Monitoring Board recommended termination of the trial due to high TB recurrence rates in the G and M regimens.

**Results:**

Of 416 patients in intent-to-treat analysis, 6 (5%) of 124, 2 (2%) of 110 and 2 (2%) of 137 patients with drug-susceptible TB in the G, M and C arms respectively had unfavorable response at the end of treatment; during the next 24 months, 17 (15%) of 115, 11 (11%) of 104 and 8 (6%) of 132 patients respectively, had TB recurrence. Of 38 drug-resistant patients 1 of 8 and 3 of 26 in the G and C arms respectively had unfavourable response at the end of treatment; and TB recurrence occurred in 2 of 7 and 2 of 23 patients, respectively. The differences in TB recurrence rates between the G and C arms was statistically significant (p = 0.02). Gastro-intestinal symptoms occurred in 23%, 22% and 9% of patients in the G, M and C arms respectively, but most reactions were mild and manageable with symptomatic measures; 1% required regimen modification.

**Conclusions:**

4-month thrice-weekly regimens of gatifloxacin or moxifloxacin with isoniazid, rifampicin and pyrazinamide, were inferior to standard 6-month treatment, in patients with newly diagnosed sputum positive pulmonary TB.

**Trial Registration:**

Clinical Trials Registry of India CTRI/2012/10/003060

## Introduction

Tuberculosis (TB) continues to be a major public health problem in much of the developing world. Even though effective anti-bacterial treatment for TB has been available for more than six decades, the long duration of treatment poses challenges to patients, who find complying with treatment over extended periods taxing. Poor compliance facilitates development of drug resistance that further aggravates the problem. Moreover, no new drug specific for TB has been introduced since the late 1960s.

Shortening the duration of treatment for TB is a global research priority. A regimen significantly shorter than the currently recommended 6-month regimen will be a boon for both patients and health care providers. Clinical trials that studied the efficacy of 3–4 month regimens in the 1970s and 1980s had high relapse rates [1]. A randomized clinical trial by the National Institute for Research in TB (NIRT) formerly the Tuberculosis Research Centre (TRC) showed that 4- or 5-month regimens containing ofloxacin (O), isoniazid (H), rifampicin (R) and pyrazinamide (Z) daily for 3 months followed by H and R twice weekly for one or two months were very effective, with 99% of patients becoming sputum culture negative at the end of treatment, and only 4% and 2% respectively suffering recurrence of TB over 24 months of follow-up [2]. This study showed for the first time that the quinolones could be advantageously used in shortening TB treatment duration.

Since then, newer fluoroquinolones, in particular moxifloxacin (M) and gatifloxacin (G) with more potent bactericidal and sterilizing activities against *mycobacteria* have emerged [3]–[6]. Both M and G have shown excellent activity *in vitro* and in animal models of TB, a favorable pharmacokinetic, and good safety profiles [6]–[9], suggesting that they may prove useful in shortening the treatment duration for TB, when used along with other first-line anti-TB drugs. Since an O-containing 4-month regimen has been shown to be very effective [2], the substitution of either M or G for O could be expected to fare even better. However, the regimen containing O that proved successful had a daily intensive phase of 3 months. Previous clinical trials have shown that regimens given three times a week can be as effective as daily regimens in the treatment of TB [1], [10], [11] while being less toxic and less costly.

We therefore studied the efficacy and safety of 4-month G or M containing regimens given thrice-weekly in patients with newly diagnosed sputum-positive pulmonary TB.

## Methodology

The protocol for this trial and supporting CONSORT checklist are available as supporting information; see Checklist S1 and Protocol S1.

### Study Participants

The study was an open label randomized controlled clinical trial conducted at Chennai and Madurai, South India, beginning in May 2004, after obtaining due regulatory approvals from the Scientific Advisory Committee and the Institutional Ethics Committee. It is registered in the Clinical Trials Registry of India (www.ctri.nic.in - CTRI/2012/10/003060). Study subjects were adult patients, 18 years or above with newly diagnosed pulmonary TB with at least 2 positive sputum cultures, and resident within a designated study area. The patients were required to consent for investigations, including screening for HIV infection, attend the health centre for supervised outpatient treatment and permit home visits. Those with previous treatment for TB exceeding 30 days, or weighing <30 kg, pregnant or lactating women and those with concomitant diabetes mellitus, severe systemic hypertension, epilepsy, serious forms of extra-pulmonary TB or HIV infection were not eligible. Written informed consent was obtained from all enrolled patients.

### Regimens and Randomisation

Eligible patients were randomly allocated to.

- 2 GHRZ_3_/2 GHR_3_ (G, H and R thrice-weekly for four months with Z for the first two months) − Gatifloxacin regimen.
- 2 MHRZ_3_/2 MHR_3_ (M, H and R thrice-weekly for four months with Z for the first two months) − Moxifloxacin regimen, or.
- 2 EHRZ_3_/4 HR_3_ (H and R thrice-weekly for six months with E and Z for the first two months) – Control regimen.

Allocation was stratified on sputum smear grading (first stratum: 0 or 1+; second stratum: 2+ or 3+) and extent of lesions in chest x-ray (≤2 zones; or >2 zones).

Restricted random allocation sequences were generated by a biostatistician using random number tables, separately for the two strata and sealed envelopes were used to assign regimens. Patients were enrolled by the physicians, and when ready for allocation, the biostatistician drew the regimen from sealed envelopes.

The medication dosages were G or M 400 mg, R 450 or 600 mg, depending on body weight (<60 kg or ≥60 kg); Z 1500 mg, and H 600 mg. All drugs were administered under direct observation as a single dose. Patients who missed clinic visits were visited at home and persuaded to attend the clinic for treatment. A maximum of 15 days was allowed for compensation of missed doses each in the intensive and continuation phase.

### Pre-treatment Investigations and Follow-up

Initial screening included four sputum specimens (two spot specimens and two overnight collections), which were examined by fluorescence microscopy [12] and cultured for *mycobacteria* in Lowenstein-Jensen (LJ) slopes by the modified Petroff's method [13]. Positive cultures were identified as *Mycobacterium tuberculosis* by standard methods [14], [15]. Drug susceptibility tests (DST) were performed on LJ media by the minimum inhibitory concentration method (for H, R, E, and O) [16]–[18]. The definitions of drug resistance for these drugs were the same as used in previous studies [2], [19], [20]; Susceptibility tests for G and M were not performed. The following tests were also done: posterior-anterior chest radiograph; electrocardiograph (ECG), urine examination for albumin, glucose, bile salts, acetyl H, and R; total and differential leucocyte counts, haemoglobin estimation, total erythrocyte count, and platelet count; liver function (bilirubin, alanine transaminase, aspartate transaminase, alkaline phosphatase); renal function (blood urea and serum creatinine); serum uric acid; random blood glucose; and ELISA for HIV antibody. The ECG was interpreted by a cardiologist.

A physician examined the patient every month and recorded adherence to treatment, any adverse drug reactions and the clinical response. Sputum specimens were examined every month by microscopy and culture, three (two overnight and one spot) during the treatment phase and two (one overnight and one spot) during the follow-up phase. One positive sputum culture was tested each month for susceptibility to H, R, E and O. Sputum specimens were given identification laboratory numbers, and bacteriological investigations were carried out by technicians who were blinded to the clinical status of the patient and the regimen. ECG was done every month. At the end of intensive phase and end of treatment a chest radiograph and blood tests comprising of hemogram, liver, renal functions and random blood sugar were done. Patients who had clinical deterioration or had adverse reactions to anti-TB drugs were examined by a team of physicians and any decision to modify the regimen was made by consultation. Patients who had a successful outcome at the end of treatment were followed up for 24 months after treatment completion.

### Outcome Measures

***Primary outcome measures*** were **a) Bacteriological status at the end of treatment,** classified as either i) “favourable”, if all three sputum cultures were negative in the last month of treatment or if one culture was positive but cultures during the subsequent months were negative without additional chemotherapy, or ii) “unfavourable”, if more than one sputum culture was positive in the last month of treatment, one of which was at least 20 colonies or more; or if treatment was changed for persistent sputum positivity, or for radiographic or clinical deterioration, or drug toxicity, or the patient died of TB during treatment, and **b) Recurrence of TB in those with a favourable response at the end of treatment.** This could be either a) Bacteriological recurrence, defined as the production of either: i) two positive sputum cultures in a 2-month period, one of which was at least 20 colonies, or ii) positive sputum cultures during four consecutive monthly examinations, none of which was 20 colonies or more or b) Clinical/radiological recurrence, defined as clinical or persistent radiological deterioration consistent with TB in the absence of bacteriological criteria defined above.

***Secondary outcome measures*** were: **a)** **Sputum culture conversion at two months,** defined as the proportion of patients who became sputum culture negative at the end of two months of treatment and **b)** **Adverse reactions to anti-TB drugs,** defined as the proportion of patients who developed adverse reactions attributable to the drugs in the treatment regimen.

### Sample Size

Applying an equivalence design and assuming that the efficacy of the control regimen to be 95% in patients with drug susceptible TB, considering both failures at the end of treatment and recurrence of TB over 24 months as the primary endpoints, with a type I error of 0.05, and a type II error of 0.20, the definition of equivalence as 5% (0.05), the approximate sample size for each regimen was calculated to be 323. Allowing for 10% attrition in patients over a 24-month follow-up period and expected proportion of patients with drug resistant TB at intake to be 10%, the final sample size was calculated to be 400 patients for each regimen.

### Statistical Analysis

The proportion of patients with various outcomes (sputum culture conversion at two months, response at the end of treatment, TB recurrence and drug adverse events) was calculated for the three regimens and the values for the test regimens were compared to that of the control regimen. The primary analyses of the outcomes of interest was both by intention-to-treat and per-protocol. Efficacy analysis was performed based on recurrences of TB in the post-treatment period of 24 months. P values less than 0.05 was considered statistically significant. The chi square test was used to compare proportions and survival analysis was done by the Kaplan-Meier method and the log-rank test was used to compare the survival distribution. Hazards ratio was calculated using Cox proportional hazards model. The statistical analyses were carried out using SPSS version 14.0.

## Results

The study design envisaged enrolling 400 patients in each arm in a 1∶1:1 ratio. However, due the non-availability of one of the test drugs (M), patients were enrolled initially in a 1∶1 ratio in the G and control regimen arms commencing in May 2004. Subsequently, when M became available (May 2005) patients were enrolled to the G, M and control regimen arms in a 1∶2:1 ratio to compensate for the delay in recruiting to the moxifloxacin arm at the onset. Later, on review of interim data, the Data and Safety Monitoring Board (DSMB) recommended termination of the G arm initially (February 2006), and later the M arm (October 2006) due to high TB recurrence rates in these two arms compared to the control regimen arm. Due to the premature termination of the study, the targeted population of 1200 patients could not be achieved.

**Figure 1:**
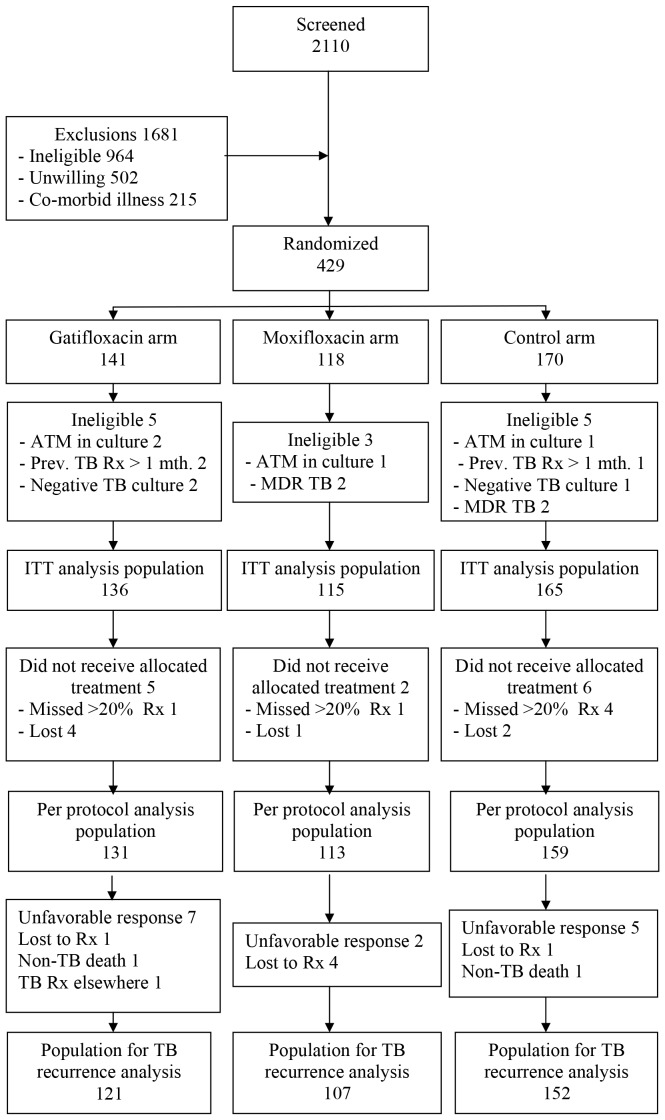
Flow diagram of patients from screening to analysis.

A total of 429 patients were admitted to the study. Of these, 13 did not fulfil the eligibility criteria (4 had sputum cultures positive for *mycobacteria* other than *M. tuberculosis*; 2 had no positive sputum cultures; 3 had received previous anti-TB treatment for more than one month; 4 had MDR-TB). The population for Intent-to-treat analysis was 416 patients (Figure 1). Of these, 6 patients missed more than 20% of treatment, or one month of treatment continuously and 7 did not have data that permitted assessment of bacteriological response at the end of treatment leaving 403 patients for per-protocol analysis.

### Intent – to – Treat Group

The baseline demographic and clinical characteristics of the 416 patients were similar among the treatment groups (Table 1). Males comprised 74%, and 72%.were aged <40 yrs. The mean body weight was 43.6 kg. Seventy nine percent each had extensive radiological involvement (>2 lung zones) or 3+ sputum cultures. Ninety one percent had bacilli susceptible to H, R E and O, and 38 had bacilli resistant to one or more drugs (27 to H, 1 to H and O, 2 to H and E, 7 to O and 1 to R).

**Table 1:** Baseline characteristics of 416 pulmonary TB patients according to regimen.

Patient characteristics	Regimen			Total patients n = 416
	Gatifloxacin n = 136	Moxifloxacin n = 115	Control n = 165	
Sex:				
Male	103 (76%)	83 (72%)	122 (74%)	308 (74%)
Female	33 (24%)	32 (28%)	43 (26%)	108 (26%)
Age (years):				
<40	90 (66%)	88 (77%)	120 (73%)	298 (72%)
≥40	46 (34%)	27 (23%)	45 (27%)	118 (28%)
Body weight (Kg.):				
Mean	43.7	44.2	43.0	43.62
Range	30.3–70.3	32.0–67.7	30.2–59.1	30.2–70.3
Sputum culture (Maximum grade):				
≤1+	4 (3%)	4 (3%)	7 (4%)	15 (4%)
2+	21 (15%)	23 (20%)	30 (18%)	74 (18%)
3+	111 (82%)	88 (77%)	128 (78%)	327 (79%)
X-ray Chest (no. of zones):				
≤2	29 (21%)	21 (18%)	38 (23%)	88 (21%)
>2	107 (79%)	94 (82%)	127 (77%)	328 (79%)
Drug susceptibility profile:				
Susceptible to H, R, E, O	128 (94%)	111 (97%)	139 (84%)	378 (91%)
Resistant to any drug	8 (6%)	4 (3%)	26 (16%)	38 (9%)
Resistant to H	5 (4%)	2 (1.7%)	20 (12%)	27 (7%)
Resistant to O	2 (2%)	–	5 (3%)	7 (1.7%)
Resistant to R	–	1 (1%)	–	1 (0.2%)
Resistant to H, E	1 (1%)	1 (1%)	–	2 (0.4%)
Resistant to H, O	–	–	1 (1%)	1 (0.2%)

### Sputum Culture Conversion (Table 2)

The proportion of patients, with negative cultures for all three monthly sputum specimens, month by month, is presented in Table 2. Culture negativity by the second month was significantly higher in the M arm (88%) compared to the control regimen arm (78%) (p = 0.04). In the G regimen arm, though the proportion of patients with negative cultures was higher (83%) compared to control regimen arm, the difference was not statistically significant (p = 0.31). the difference in culture negativity between the M and G arms was also not statistically significant (p = 0.29).

**Table 2:** Sputum culture negativity based on three specimens each month, among 416 patients.

Month of treatment	Regimen								
	Gatifloxacin (n = 136)			Moxifloxacin (n = 115)			Control (n = 165)		
	No. examined	Culture negative		No. examined	Culture negative		No. examined	Culture negative	
		n	%		n	%		n	%
1	135	34	25	113	37	33	165	46	28
2	133	110	83	112	98	88	164	128	78
3	130	125	96	112	111	99	162	157	97
4	129	123	95	113	112	99	163	156	96
5	–	–	–	–	–	–	162	153	94
6	–	–	–	–	–	–	163	155	95

### Status at the End of Treatment (Table 3)

Table 3 describes the status at the end of treatment according to the initial drug susceptibility profile in 409 of 416 patients since the response could not be assessed in 7 patients. Ninety five percent of those in the G arm and 98% of those in the M arm and 97% of those in the control arms had a favourable bacteriological status at the end of treatment. Seven patients in the G arm, 2 in the M arm and 5 in the control regimen arm had an unfavourable outcome.

**Table 3:** Status at end of treatment in 416 patients according to initial drug susceptibility profile (Intent – to –treat analysis).

Regimen	Initial sputum cultures susceptible to H, R, E and O			Initial sputum cultures resistant to one or more drugs			All patients		
	Total patients	Response at end of treatment		Total patients	Response at end of treatment		Total patients	Response at end of treatment	
		Favourable	Unfavourable		Favourable	Unfavourable		Favourable	Unfavourable
		n (%)	n (%)		n (%)	n (%)		n (%)	n (%)
Gatifloxacin	124*	118 (95)	6 (5)	8	7	1 (1)	132*	125 (95)	7∧ (5)
Moxifloxacin	110*	108 (98)	2 (2)	4	4	0 (0)	114*	112 (98)	2@ (2)
Control	137*	135 (98)	2 (2)	26	23 (88)	3 (12)	163*	158 (97)	5^$^ (3)

* Response could not be assessed in 7 lost patients – Gatifloxacin (4), Moxifloxacin (1), Control (2).

∧ 4 patients –Treatment changed for toxicity (Seizure - 3, Cardiac –1), unfavouable bacteriological response –3.

@ Treatment changed for pneumothorax −1, unfavouable bacteriological response −1.

$ Treatment changed for hepatotoxicity –1, unfavouable bacteriological response −4.

Of the 10 patients with drug susceptible TB (6, 2 and 2 in the G, M and control regimen arms respectively) who had an unfavourable response at the end of treatment, one patient each in the M arm and control regimen arm developed H resistance. Of the 38 patients with resistance to one or more drugs (8, 4 and 26 in the G, M and control regimen arms respectively), one patient in the G arm and 3 in the control regimen arm had an unfavourable outcome at the end of treatment. One of the 3 patients in the control regimen arm who had initial H resistance developed additional resistance to R.

### Bacteriological Recurrence (Table 4, Figure 2)

Of the patients who had a favourable response at the end of treatment (125, 112 and 158 in the G, M and control regimen arms respectively), all but 3, 4 and 3 patients were followed-up for 24 months post-treatment. During this period 16%, 10% and 6% of patients in the G, M and control regimen arms respectively had recurrence of TB. All but one of these were pulmonary TB and one patient developed TB lymphadenitis. Among those with drug resistant TB, 2 of 7 in the G arm and 2 of 23 in the control regimen arm had recurrence of TB. The drug susceptibility patterns of the cultures were consistent with pre-treatment profiles in all the patients with bacteriological recurrence.

**Table 4:** TB recurrence during 24 months of post-treatment follow-up in 395 patients according to regimen and initial drug susceptibility profile (Intent-to-treat analysis).

Regimen	Initial Drug susceptibility profile	Total Patients	Lost to follow-up			Patients assessed for TB recurrence	TB recurrence	Month of TB recurrence (post treatment)		
			Non-TB death	Default	Others		n (%)	1–6	7–12	13–19
Gatifloxacin	All patients	125	1	1	1	122	19 (16)	19	0	0
	Susceptible	118	1	1	1	115	17 (15)	17	0	0
	Resistant	7	0	0	0	7	2 (29)	2	0	0
Moxifloxacin	All patients	112	0	4	0	108	11 (10)	9	1	1
	Susceptible	108	0	4	0	104	11 (11)	9	1	1
	Resistant	4	0	0	0	4	0 (0)	0	0	0
Control	All patients	158	1	2	0	155	10 (6)	8	0	2
	Susceptible	135	1	2	0	132	8 (6)	6	0	2
	Resistant	23	0	0	0	23	2 (9)	2	0	0

The differences in the TB recurrence rate between the G arm and the control arm was statistically significant (16% vs 6%; RR 0.90; 95% CI 0.83–0.98; p = 0.02). The difference in the recurrence rate between the M arm (10%) and the control arm (6%) was not statistically significant (RR 0.96; 95% CI 0.89–1.04; p = 0.38). The difference in the recurrence rate between the G and M arms was also not statistically significant. (RR 0.94; 95% CI 0.85–1.04; p = 0.31).

**Figure 2:**
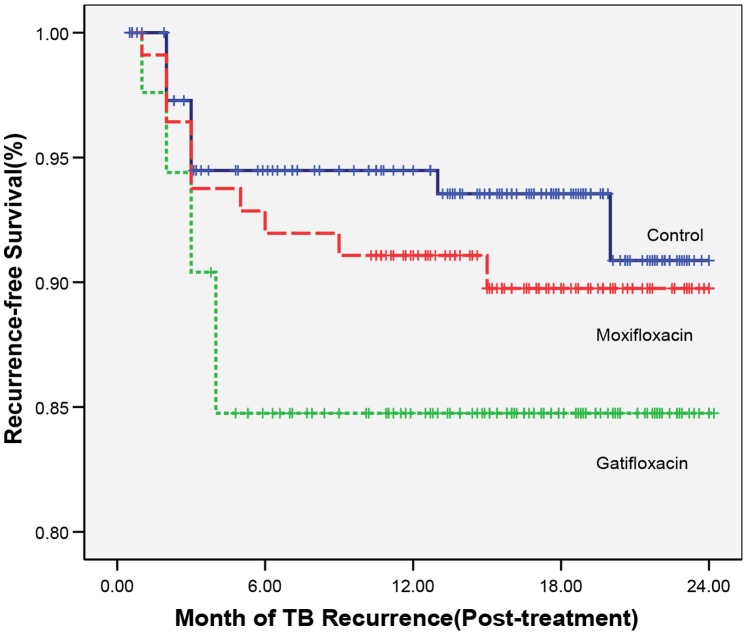
Kaplan – Meier analysis of post-treatment TB recurrence-free survival over 24 months in the study regimens.

All the 19 TB recurrences in the G arm, 9 of the 11 recurrences in the M arm and 8 of 10 recurrences in the control regimen arm occurred within 6 months of stopping treatment. A time-to-event analysis using the Kaplan-Meier method for post-treatment TB recurrence in the three study regimens over 24 months is shown in Figure 2. Cox proportional hazards analysis showed a hazard ratio of 2.26 (95% CI 1.05–4.87; p = 0.04) and 1.41 (95% CI 0.60–3.32; p = 0.432) for the G and M arms respectively compared to the control regimen arm.

### Adverse Reactions to Anti-TB Drugs (Table 5)

Symptoms attributable to anti-TB drugs in the 416 patients is described in Table 5. The commonest adverse events were gastro-intestinal symptoms (nausea, vomiting, abdominal discomfort) which occurred in 23%, 22% and 9% in G, M and control regimen arms respectively. Giddiness or dizziness was observed in 18%, 15% and 3% respectively. Arthralgia attributable to Z was seen in 4%, 3% and 2% of patients in the three treatment arms; Cutaneous rash occurred in 4%, 4% and 3% respectively. Anti-TB drugs were withheld and re-introduced for one patient in the G regimen arm for vomiting. Jaundice occurred in 1 patient in the control regimen arm for whom treatment was modified and 1 patient had peripheral neuropathy. Three patients had seizures; all in the G regimen arm for whom the drug was terminated. Two patients had prolongation of QTc interval in the ECG; one each in the G (in whom the drug was terminated) and M (at the end of treatment) regimen arms. Dysglycemia was not seen in any patient.

**Table 5:** Adverse reactions attributable to anti -TB drugs in 416 patients (Intent- to – treat group).

Regimen	Drug adverse reactions								
	Patients	Arthralgia	Cutaneous	Giddiness	Gastro-intestinal	Hepatic	Cardiac	Seizures	Peroipheral neuropathy
	(n)	n (%)	n (%)	n (%)	n (%)	n (%)	n (%)	n (%)	n (%)
Gatifloxacin	136	6 (4)	6 (4)	24 (18)	31 (23)	0 (0)	1 (1)*	3 (2)*	0 (0)
Moxifloxacin	115	3 (3)	5 (4)	17 (15)	25 (22)	0 (0)	1(1)	0 (0)	0 (0)
Control	165	4 (2)	5 (3)	5 (3)	15 (9)	1 (1)@	0 (0)	0 (0)	1 (1)

* Gatifloxacin terminated (Seizure –3, cardiac −1).

@ Treatment changed for heptotoxicity −1.

### Per – Protocol Analysis

Per-protocol analysis was done in 403 patients (Table 6). Six patients who missed more than 20% of treatment, or one month of treatment continuously and 7 patients who did not have data that permitted assessment of bacteriological response at the end of treatment were excluded.

**Table 6:** Status at end of treatment in 403 patients according to initial drug susceptibility profile (Per-protocol analysis).

Regimen	Initial Drug susceptibility profile	Total patients (n)	Favourable response	Unfavourable response	
				Bacteriological	Treatment changed
			n (%)	n (%)	n (%)
Gatifloxacin	All patients	131	124 (95)	3 (2)	4 (3)#
	Susceptible	123	117 (95)	2 (2)	4 (3)#
	Resistant	8	7	1	0
Moxifloxacin	All patients	113	111 (98)	1 (1)	1 (1)@
	Susceptible	109	107 (98)	1 (1)	1 (1)@
	Resistant	4	4	0	0
Control	All patients	159	154 (97)	4 (2)	1 (1)#
	Susceptible	133	131(98)	1 (1)	1 (1)#
	Resistant	26	23 (88)	3 (11)	0

# Treatment changed for toxicity.

@ Treatment changed for pneumothorax -1.

Of the 403 patients, 365 had bacilli susceptible to H, R, E and O, and 38 had bacilli resistant to one or more drugs (27 to H, 1 to H and O, 2 to H and E, 7 to O and 1 to R).

Essentially, the response at the end of treatment was very similar to what was observed in the intent-to-treat analysis; viz. 95%, 98% and 97% in the G, M and control regimen arms respectively had a favourable response; 7, 2 and 5 patients had an unfavourable response.

Table 7 describes recurrence of TB in the per-protocol analysis. This was similar to what was observed in the intent-to-treat analysis. Recurrence of TB occurred in 16%, 10% and 7% in the G, M and control regimen arms respectively.

**Table 7:** TB recurrence during 24 months of post-treatment follow-up in 389 patients according to regimen and initial drug susceptibility profile (Per-protocol analysis).

Regimen	Initial Drug susceptibility profile	Totalpatients	Lost to follow-up			Patients assessed for TB recurrence	TB recurrence	Month of TB recurrence (post treatment)		
			Non-TB death	Default	Others		n (%)	1–6	7–12	13–19
Gatifloxacin	All patients	124	1	1	1	121	19 (16)	19	0	0
	Susceptible	117	1	1	1	114	17 (15)	17	0	0
	Resistant	7	0	0	0	7	2	2	0	0
Moxifloxacin	All patients	111	0	4	0	107	11 (10)	9	1	1
	Susceptible	107	0	4	0	103	11 (11)	9	1	1
	Resistant	4	0	0	0	4	0	0	0	0
Control	All patients	154	1	1	0	152	10 (7)	8	0	2
	Susceptible	131	1	1	0	129	8 (6)	6	0	2
	Resistant	23	0	0	0	23	2	2	0	0

## Discussion

The study was designed based on the encouraging results from an earlier study with a 4-month ofloxacin containing regimen with an initial daily intensive phase and the desire to develop a fully intermittent regimen of similar duration. Since previous experience with TB treatment trials had suggested that fully intermittent regimens may be as effective as daily regimens while at the same time being less toxic and less expensive, we were hopeful of a favourable outcome. Contrary to our expectations, while the results at the end of treatment were comparable, the TB recurrence rates in the gatifloxacin and moxifloxacin regimens were higher compared to the control regimen, necessitating the premature termination of the trial. However, this truncated trial still yielded important information that we considered worth communicating and that might guide future research in this area.

We found that the response at the end of treatment was uniformly high in all 3 regimens, with 95% and 98% of the patients treated with a thrice-weekly 4-month gatifloxacin and moxifloxacin regimens respectively becoming culture negative at the end of treatment, compared to 97% in the control regimen. This is in keeping with the previous experience at the NIRT when 99% of patients treated with the ofloxacin containing 4-month regimen, with a daily intensive phase were culture negative at the end of treatment [2]. Even among those with initial drug resistance, none of the 4 patients treated with the moxifloxacin regimen had an unfavourable outcome while one of 8 patients treated with the gatifloxacin regimen and 3 of the 26 patients treated with the control regimen had an unfavourable response. Only one of 21 patients with initial H resistance treated with the control regimen developed MDR TB.

The proportion of sputum culture conversion to negative at 2 months is an important parameter for assessing the efficacy of a TB drug regimen [1]. In this study, 83% and 88% of patients treated with the gatifloxacin and moxifloxacin regimens respectively became sputum culture negative at 2 months compared to 78% of those treated with the control regimen. These culture conversion rates were significantly lower than the 92–94% culture negativity that was observed in our previous ofloxacin study with daily dosing [2].

The most striking finding of this study was that the recurrence rate of TB during 24 months of post-treatment follow-up was higher in the gatifloxacin arm (16%) compared to the moxifloxacin (10%) and control regimen arms (6%). The recurrence rates in both the quinolone regimens was much higher than the 4% recurrence in our previous study in which OHRZ was given daily for 3 months followed by RH twice weekly. [2] Clearly, a thrice weekly 4-month regimen is inferior to a 4-month regimen with an initial daily phase in terms of recurrence of TB. However, even though the recurrence rate in the moxifloxacin arm (10%) was higher than that in the control regimen arm (6%), the difference was not statistically significant. It is pertinent to point out that 90% of patients treated with the 4-month thrice-weekly moxifloxacin regimen were recurrence free 24 months after treatment completion.

The majority of the TB recurrences (all in gatifloxacin arm and 9 of 11 in the moxifloxacin arm) occurred within 6 months of stopping treatment. This is in keeping with previous experience [2], [21]. While this suggests that these recurrences were probably true relapses rather than re-infections, in the absence of genotying it is difficult to be sure. In contrast to this, two of the 10 recurrences in the control regimen arm, i.e. 20%, occurred beyond 12 months of follow-up compared to none in the gatifloxacin arm and 1 of 11 in the moxifloxacin arm. Again it is difficult to say whether these could represent re-infection rather than re-activation.

In analysing the possible reasons for the high TB recurrence rates in the two quinolone regimens, we need to consider the following issues: (a) dosage of the quinolone – we used the standard recommended daily dosage of 400 mg for both gatifloxacin and moxifloxacin, even though both drugs were administered thrice weekly. It is possible that this dosage was insufficient in terms of pharmacokinetic effects when administered thrice weekly; (b) rhythm of drug administration - intermittent drug administration in TB is based on the phenomenon of lag phase or the post antibiotic effect (PAE) exhibited by the anti-TB drugs [22]. An in-vitro study has shown that moxifloxacin has no PAE [23]; which could be the reason for its poor performance when given thrice- weekly throughout the treatment period. On the other hand, it has been shown in an animal study that moxifloxacin has excellent sterilizing activity when given once-weekly along with H and rifapentine [4]. This needs to be evaluated in human studies.

Both the quinolone regimens were well tolerated. While gastrointestinal symptoms and giddiness were significantly more common in those treated with the quinolone regimens, most of these were mild and were managed with symptomatic treatment Three of 136 patients treated with the gatifloxacin regimen developed seizures. Though 2 patients (one each in the gatifloxacin and moxifloxacin arms) had marginal prolongation of the QTc interval in the ECG neither had adverse consequences. Significantly none of the patients developed dysglycemia, which has been recognized as a known side effect of gatifloxacin [24], even though blood sugar levels were not closely monitored and was done only during routine monthly check-up.

This analysis of a truncated study has many limitations. Case recruitment could not proceed as planned due to operational reasons, and occurred in a staggered manner, initially to the gatifloxacin and control regimen arms, and later to the three arms of the study. The targeted sample size could not be completed due to the premature termination of the study on the recommendation of the DSMB. Even though the primary outcome of our study was defined as a combination of the results at the end of treatment and TB recurrence in those with a favourable treatment outcome, the DSMB recommendation to terminate patient recruitment, first to the gatifloxacin regimen and later to the moxifloxacin regimen was based only on the post-treatment TB recurrence rates. We did not do genotyping to distinguish between reactivation and re-infection. Even though these issues limit the robustness of the analysis, the data presented in this report still gives valuable information that can guide future research, especially in view of the current interest in the role of quinolones in shortening TB treatment duration. What appears to be clear is that while a 4-month regimen including a quinolone in addition to other bactericidal anti-TB drugs H, R and Z with at least initial daily phase of 3 months was very successful in the treatment of sputum positive patients with pulmonary TB [2], a regimen of similar duration but given 3 times a week throughout (in spite of having a quinolone throughout the 4 months) was clearly inferior. Perhaps the search for a shorter TB regimen using quinolones should necessarily include a daily phase at least initially. Currently at the NIRT a clinical trial is in progress to study the efficacy and safety of 3 and 4-month moxifloxacin-containing regimens with a daily intensive phase for the treatment of patients with sputum positive pulmonary TB (CTRI/2008/091/000024). Two multicentre global phase III studies with gatifloxacin and moxifloxacin containing regimens in patients with sputum positive pulmonary tuberculosis are also in progress [25], [26]. Results from these three clinical trials will ultimately determine whether a 4-month treatment regimen will be successful in treating patients with sputum positive pulmonary TB.

## Supporting Information

Protocol S1 **Study Protocol.**
(DOC)

Checklist S1 **CONSORT Checklist.**
(DOC)
