# Supplementary material for: Randomized Clinical Trial of Thrice-Weekly 4-Month Moxifloxacin or Gatifloxacin Containing Regimens in the Treatment of New Sputum Positive Pulmonary Tuberculosis Patients
Source: PLoS One. 2013 Jul 3;8(7):e67030. doi: 10.1371/journal.pone.0067030 (PMC3700922; doi:10.1371/journal.pone.0067030)
Supplement: Protocol S1 — Study Protocol. (DOC) [file pone.0067030.s001.doc]

Study Protocol S1

27^th^ December 2003

A STUDY OF THE EFFICACY AND TOLERABILITY OF MOXIFLOXACIN AND GATIFLOXACIN CONTAINING REGIMENS IN THE TREATMENT OF PATIENTS WITH SPUTUM-POSITIVE PULMONARY TUBERCULOSIS

**Background:**

Many randomized clinical trials have established the efficacy of highly effective regimens for the treatment of patients with drug-susceptible tuberculosis; however, they must be administered for a minimum of six months to achieve optimal results. [1]. Non-adherence to treatment remains a major problem even with these six-month regimens. To address this, directly observed therapy (DOT) has been advocated as a standard of care worldwide and is a vital component of the WHO’s global strategy for TB control. [2]. The Revised National Tuberculosis Control Programme (RNTCP) of the Government of India is based on the global strategy of WHO. However, the administrative burden of providing DOT for all patients is considerable and the RNTCP recommends full DOT only for the initial intensive phase of treatment and partial DOT for the continuation phase. Thus, there is a need for new drugs that would permit significant shortening of treatment.

The demonstration of anti-mycobacterial activity of the fluoroquinolone group of drugs has opened a window of opportunity to address this particular need for the shortening of the duration of treatment. [3,4] A randomized clinical trial by the TRC has shown that by using ofloxacin in the intensive phase of a 4-month regimen, it is feasible to achieve a cure rate of 99% at the end of treatment and a relapse rate of less than 5% over 24 months of post-treatment follow-up in patients with drug susceptible bacilli. [5] Since then, data has emerged about newer fluoroquinolones, in particular moxifloxacin, gatifloxacin, levofloxacin, sparfloxacin, clinafloxacin, lomefloxacin, and others, which have more potent anti-mycobacterial activity both in terms of bactericidal activity and sterilizing activity. [6,7,8]

Of the currently available fluoroquinolones, moxifloxacin and gatifloxacin have been shown to have excellent activity *in vitro* and in animal models of tuberculosis, a favorable pharmacokinetic profile (serum half-life of 10-12 hours), and excellent safety profiles. However, these drugs have not been evaluated for tuberculosis treatment and have only been formally evaluated in treatment durations of 2 week or less for acute bacterial respiratory infections such as community acquired pneumonia and acute exacerbations of chronic sinusitis. Therefore, there is a need to evaluate the safety of more prolonged dosing of moxifloxacin and gatifloxacin and to assess their activity against clinical tuberculosis.

Moxifloxacin is a new 8-methoxy fluoroquinolone antibiotic with excellent activity against *Mycobacterium tuberculosis* both *in vitro* and in animal models. The MIC for clinical isolates of *Mycobacterium tuberculosis* ranges from 0.12 to 0.5 ug/ml and it is the most active among the newer set of fluroquinolones. [9,10,11] In an experimental study on female Swiss mice infected with H37Rv and treated with various drug dosages, moxifloxacin was more potent than sparfloxacin and clinafloxacin, at the 100 mg/kg dosage comparable to that of isoniazid.

The recommended dosage of moxifloxacin is 400 mgm.

Gatifloxacin is a new broad-spectrum fluoroquinolone which is active against gram-positive, gram-negative and anaerobic organisms and has been used for the treatment of acute bacterial exacerbation of chronic bronchitis, acute sinusitis, community-acquired pneumonia, uncomplicated and complicated urinary tract infections, pyelonephritis and uncomplicated gonorrheal infections.

Quinolones inhibit DNA gyrase and topoisomerase IV. DNA gyrase is an essential enzyme that is involved in the replication, transcription and repair of bacterial DNA. Topoisomerase IV is an enzyme known to play a key role in the partitioning of the chromosomal DNA during bacterial cell division.

Gatifloxacin is well absorbed from the gastrointestinal tract after oral administration and can be given without regard to food. The absolute bioavailability of gatifloxacin is 96%, and peak plasma concentrations usually occur in one to two hours. Mean steady-state peak and trough plasma concentrations attained following a dosing regimen of 400 mg once daily are approximately 4.2 µg/mL and 0.4 µg/mL, respectively, for oral administration and 4.6 µg/mL and 0.4 µg/mL, respectively, for IV administration.

Gatifloxacin undergoes limited biotransformation in humans. In vitro studies with cytochrome P450 isoenzymes indicate that gatifloxacin neither inhibits CYP3A4, CYP2D6, CYP2C9 or CYP1A2, nor induces hepatic enzymes. Therefore, it is unlikely that gatifloxacin will alter the metabolic elimination of itself or other drugs.

Gatifloxacin undergoes both glomerular filtration and tubular secretion and greater than 70% is excreted as unchanged drug.

The recommended dosage of gatifloxacin is 400 mgm.

Since gatifloxacin and moxifloxacin have anti-mycobacterial activity superior to that of ofloxacin and their safety profile have been shown to be excellent, albeit when used for short duration (up to 14 days), they may prove to be useful drugs to shorten treatment duration for tuberculosis, when used along with other first-line anti-TB drugs. Since an ofloxacin-containing 4-month regimen has been shown to be highly effective, the substitution of either moxifloxacin or gatifloxacin for ofloxacin could be expected to fare even better.

It is therefore proposed to carry out a study to assess the tolerability of moxifloxacin and gatifloxacin containing regimens in patients with smear-positive pulmonary tuberculosis with particular reference to their long term use, and to assess the efficacy of these regimens in terms of culture conversion at 2 months, results at the end of treatment and relapse rates up to 24 months of follow-up.

**Setting:**

The study will be carried out by the Tuberculosis Research Centre in Chennai and Madurai, in collaboration with our partners, the Institute of Thoracic Medicine, RNTCP Clinics of the Corporation of Chennai, Government Thiruvatteeswarar Hospital, the Ramakrishna Mutt Charitable Dispensary, the Chest Clinics of the Government Royapettah Hospital, the Government Dispensary Saidapet, all in Chennai, and the Chest Clinic of the Rajaji Hospital in Madurai.

**Patients:**

Patients aged 18 years and above residing in or around Chennai or Madurai will be eligible for enrollment to the study. They should not have anti-TB treatment in the past or should have had less than one month of treatment (but less than one week in the preceding one month before enrollment in the study).

They should have sputum culture-positive pulmonary tuberculosis (at least two cultures should be positive). Patients will be enrolled to the study when two sputum smears are positive and will be retained for analysis only if two cultures are positive.

They should consent to attend the treatment centre for supervised treatment for 6 months and for home visits by the staff of the center.

They should give written informed consent.

The following patients will not be eligible for the study:

Those with

- body weight less than 30 kg
- hepatic or renal disease as evidenced by clinical or biochemical abnormalities
- diabetes mellitus
- a history of seizures
- psychiatric illness
- an abnormal electrocardiogram or those on anti-arrhythmic medication
- Those in a moribund state
- Those sero-positive for HIV antibodies
- Pregnant or lactating women

**Study design:**

This will be a randomised multi-centric clinical trial

**Treatment Regimens:**

Patients found eligible for admission to the study will be randomly allocated to the following regimens:

Regimen 1:

2 MHRZ thrice weekly/ 2 MHR thrice weekly: moxifloxacin, isoniazid, rifampicin and pyrazinamide thrice-weekly for 2 months followed by moxifloxacin, isoniazid and rifampicin thrice-weekly for 2 months. (Duration 4 months).

Regimen 2:

2 GHRZ thrice weekly/ 2 GHR thrice weekly: gatifloxacin, isoniazid, rifampicin and pyrazinamide thrice-weekly for 2 months followed by gatifloxacin, isoniazid and rifampicin thrice-weekly for 2 months. (Duration 4 months).

Regimen 3:

2 EHRZ thrice weekly/ 4 HR thrice weekly: ethambutol, isoniazid, rifampicin and pyrazinamide thrice-weekly for 2 months followed by isoniazid and rifampicin thrice-weekly for 4 months. (Duration 6 months)

Patients allocated to regimens 1 and 2 will receive 27 doses each in the intensive and continuation phase respectively. Missed doses will be compensated up to maximum of 15 days after the scheduled 2^nd^ and 4^th^ monthly examinations.

Patients allocated to regimen 3 will receive 27 doses in the intensive phase and 54 doses in the continuation phase. Missed doses will be compensated up to a maximum of 15 days after the scheduled 2^nd^ and 6^th^ monthly examinations respectively.

Every dose of treatment will be given under direct observation. Patients who default for treatment will be visited at home by a Health Visitor, Social Worker and Doctor sequentially, and motivated to attend for treatment.

Dosage of drugs in mg:

Isoniazid - 600; rifampicin - 450; pyrazinamide - 1500; ethambutol - 1200; gatifloxacin - 400; moxifloxacin – 400

Patients weighing 60 kg or more will receive rifampicin 600 mg

**Sample size:**

Applying an equivalence design and

assuming that the efficacy of the control regimen (Regimen 3) will be 95% (0.95) in patients with drug susceptible disease, taking into account both failures at the end of treatment and relapse over 24 months,

Type I error to be 5% (0.05, 1 tail) Z = 1.64 and

Type II error to be 10% (0.1) Z = 1.28 and

the definition of equivalence as 5% (0.05),

the sample size required for each regimen was calculated to be 2 (0.95 x 0.05) (1.64 + 1.28)2/(0.05)^2^ = 323.

Sample size for 3 regimens = 3 x 323 = 969

Loss in follow-up over 24 months is expected to be 10% (0.01)

Proportion of patients with drug resistant tuberculosis at intake is expected to be 10% (0.01)

The required sample size for the trial will therefore be 969/ (0.9 x 0.9) = 1196 or approximately 1200 patients

**Study Progress Review Panel:**

A Panel consisting of three doctors, a bacteriologist and a statistician will review:

- all cases at the end of treatment after the sputum smear results are available,
- all cases with sputum cultures resistant to rifampicin and isoniazid,
- change of treatment on clinical/ or radiological deterioration,
- bacteriological relapse,
- at any other time if considered necessary by the doctors.

Patients will be prescribed calcium lactate one tablet daily after treatment phase till the end of follow-up

**Outcome measures:**

The following outcome measures will be compared between regimens:

a) Sputum culture conversion at 2 months of treatment,

b) Favourable bacteriological response at end of treatment

c) Unfavourable bacteriological response at end of treatment

d) Doubtful bacteriological response at end of treatment

e) Relapse after successful treatment.

f) Adverse reactions to anti-TB drugs, especially to moxifloxacin and gatifloxacin.

**Definition of Outcome Measures:**

*Sputum culture conversion at 2 months*: The proportion of patients with negative sputum cultures at 2 months of treatment.

*Favourable bacteriological response at end of treatment:*

A patient will be classified as having had a 'Favourable' bacteriological response at end of treatment if all three cultures are negative in the last month of treatment.

*Unfavourable bacteriological response:*

A patient will be classified as having had an 'Unfavourable' bacteriological response at end of treatment if more than one culture is positive in the last month of treatment, one of which is at least 20 colonies or more.

*Doubtful bacteriological response:*

A patient will be classified as having had a 'Doubtful' bacteriological response if one or more cultures are positive in the last month of treatment, none of which is 20 colonies or more.

The physicians acting in consultation can decide on a change of treatment based on clinical and/or radiological deterioration and persistent sputum positivity. Such cases will be put up to the Panel for ratification of the decision.

*Relapse after treatment:*

In patients who have had a favourable or doubtful bacteriological response at the end of treatment, relapse during follow-up is defined as either I) two positive sputum cultures in a 2-month period, one of which is at least 20 colonies, (such patients will be retreated if at least one sputum smear is also positive)

or ii) positive sputum cultures during four consecutive monthly examinations, one of which is at least 20 colonies.

**Clinical assessments and Investigations:**

*Baseline:*

History of present complaints and past medical history.

Detailed clinical examination including testing for aquity of vision and colour vision.

Detailed history of previous treatment for tuberculosis and history of use of quinolones and anti-arrhythmic medication. All available prescriptions slips will be scrutinized and recorded.

Assessment by a social worker for domestic stability, family history, occupational history and sociological suitability for likely adherence to treatment protocol.

Home visit by a social worker and health visitor.

At least four sputum specimens will be examined by smear (fluorescent microscopy) and culture (with LJ medium) for *Mycobacterium tuberculosis*. Positive cultures will be identified by catalase production at 68°C and PNB test. Drug susceptibility tests for streptomycin, isoniazid, rifampicin, ethambutol, moxifloxacin and gatifloxacin will be done on positive cultures.

X-ray chest PA view

Urinalysis for albumin, sugar, bile pigments, acetyl isoniazid, rifampicin and for cytology.

ECG in 12 leads. The ECGs will be interpreted by a cardiologist.

Total and differential white cell counts, red blood cell count, haemoglobin estimation, platelet count.

Random blood sugar, blood urea, serum creatinine, serum uric acid, serum bilirubin, serum alanine and aspartate transaminase, serum alkaline phosphatase and gamma glutamy transaminase (GGT).

ELISA for HIV antibodies.

*During treatment:*

Every month during the treatment phase the following will be done:

Clinical examination

Weight will be recorded

Monitoring for adverse reactions to drugs, especially moxifloxacin and gatifloxacin.

Three sputum specimens will be examined by smear (fluorescent microscopy) and culture (LJ medium) for *Mycobacterium tuberculosis* and identification and drug susceptibility tests for isoniazid, rifampicin, ethambutol, moxifloxacin and gatifloxacin will be carried out on positive cultures.

ECG in 12 leads

Urinalysis will be done for albumin and cytology

X-ray Chest PA view at the 2^nd^ monthly examination and at the end of treatment

Total and differential white cell counts, red blood cell count, haemoglobin estimation and platelet count at the time of the 2^nd^ and 4^th^ monthly examinations.

Blood sugar, blood urea, serum creatinine, serum uric acid, serum bilirubin, serum alanine and aspartate transaminase, serum alkaline phosphatase and gamma glutamy transaminase (GGT) at the time of the 2^nd^ and 4^th^ monthly examinations.

*During follow-up:*

After treatment is completed all patients will be followed up every month for 24 months (28 to 30 months from start of treatment). During these monthly visits the following evaluations/ investigations will be done:

Clinical examination

Weight will be recorded

Two sputum specimens will be examined by smear (fluorescent microscopy) and culture (LJ medium) for *Mycobacterium tuberculosis* and identification and drug susceptibility tests for isoniazid, rifampicin, ethambutol, moxifloxacin and gatifloxacin will be carried out on positive cultures.

Urinalysis will be done for albumin, acetyl isoniazid, rifampicin and cytology.

In addition,

X-ray Chest PA view will be done at the 12^th^ monthly examination and at the end of treatment.

**Follow-up:**

Patients will be followed up for 24 months after treatment completion (28 to 30 months from start of treatment). They will be discharged from the study after the final month culture results are negative.

**Funding:**

The study will be funded solely by the Tuberculosis Research Centre

**Ethical Considerations:**

Patients will be informed in their mother tongue or a language that they understand, about the purpose of the study and the investigations that will be carried out to make a diagnosis and to monitor the response to treatment, and the frequency with which they will have to attend the treatment centre for supervised treatment. They will be informed of the drugs that will be used to treat them and the side effects that may occur due to the drugs. A Patient Information Sheet will be made available to the patient. Their consent will be obtained with reference to the investigations that will be carried out, the frequency of their attendance at the treatment centre, and their willingness to permit visits to their homes. Counseling will be done and a separate consent obtained before drawing blood for HIV antibody testing.

Any patient who does not respond to the treatment regimen and those who relapse during the period of follow-up will be treated with an appropriate regimen.

All diagnostic procedures and treatment will be entirely free for the patients.

Patients will be informed of their right to withdraw from the study at any time, without any penalty, if they so desired.

*Age of participants*:

There have been reports of the effect of fluoroquinolones on cartilage in animal experiments. Keeping this in view we had recruited only adult patients for the TRC ofloxacin studies. In this study also we will confine the study subjects to adults of 18 years and above, till there is categorical proof that moxifloxacin and gatifloxacin are safe for children, and do not affect bone growth.

**REFERENCES**

1. Fox W, Ellard GA, Mitchison DA. Studies on the treatment of tuberculosis undertaken by the British Medical Research Council Tuberculosis Units, 1946-1986, with relevant subsequent publications. Int J Tuberc Lung Dis 1999;3:S231- S279
2. World Health Organization. Global tuberculosis control. Geneva, Switzerland: World Health Organization, 2001
3. Kennedy N, Fox R, Kisyombe GM, et al. Early bactericidal and sterilizing activity of ciprofloxacin in pulmonary tuberculosis. Am Rev Respir Dis 1993;148:1547-51
4. Daniel Herbert, Paramasivan CN, Venkatesan P, Kubendran G, Prabhakar R and Micheson DA. Evaluation of bactericidal action of ofloxacin and sulbactum-ampicillin alone and in combination with rifampicin and isoniazid on *M tuberculosis in vitro*. Anti-microb. Agents Chemother 1996; 40: 2296-99
5. Tuberculosis Research Centre. Shortening short course chemotherapy. Ind J Tuberc 2002; 49: 27-38
6. Sirgel FA, Donald PR, Odhiambo J, et al. A multicentre study of the early bactericidal activity of anti- tuberculosis drugs. J Antimicrob Chemother 2000; 45(6)**:** 859-70
7. Lounis N, Bentoucha A, Truffot-Pernot C, et al. Effectiveness of once-weekly rifapentine and moxifloxacin regimens against *Mycobacterium tuberculosis* in mice. Antimicrob Agents Chemother 2001; 45(12)**:** 3482-6;
8. Hu Y, Coates AR, Mitchison DA. Sterilizing activities of fluoroquinolones against rifampin-tolerant populations of *Mycobacterium tuberculosis.* Antimicrob Agents Chemother 2003; 47(2): 653-7).
9. Woodcock JM, Andrews JM, Boswell FJ, Brenwald NP, Wise R. *In vitro* activity of BAY 12-8039, a new fluoroquinolone. Antimicrob Agents Chemother 1997; 41(1)**:** 101-6
10. Gillespie SH, Billington O. Activity of moxifloxacin against mycobacteria. J Antimicrob Chemother 1999; 44(3)**:** 393-5
11. Rodriguez JC, Ruiz M, Climent A, Royo G. *In vitro* activity of four fluoroquinolones against *Mycobacterium tuberculosis*. Int J Antimicrob Agents 2001;17(3)**:** 229-31

25^th^ October 2005

Amendment to protocol entitled

A STUDY OF THE EFFICACY AND TOLERABILITY OF MOXIFLOXACIN AND GATIFLOXACIN CONTAINING REGIMENS IN THE TREATMENT OF PATIENTS WITH SPUTUM-POSITIVE PULMONARY TUBERCULOSIS

dated 27^th^ December 2003

Page 5: Patients: under “the following patients will not be eligible for the study” include “those with defective vision”.

Page 5: Treatment Regimens:

Lines 15 and 16:

In place of “Patients allocated to regimens 1 and 2 will receive 27 doses each in the intensive and continuation phase respectively” amended as “Patients allocated to regimens 1 and 2 will receive 26 doses each in the intensive and continuation phase respectively”.

Page 5: Treatment Regimens:

Lines 18 and 19:

In place of “Patients allocated to regimen 3 will receive 27 doses in the intensive phase and 54 doses in the continuation phase: amended as “Patients allocated to regimen 3 will receive 26 doses in the intensive phase and 52 doses in the continuation phase”.

Page 7: Definition of Outcome Measures:

Doubtful bacteriological response:

Line 13:

After the sentence “A patient will be classified as having had a 'Doubtful' bacteriological response if one or more cultures are positive in the last month of treatment, none of which is 20 colonies or more” add “or has one culture positive in the last month of treatment”.

Page 8: Clinical assessments and Investigations:

*Baseline:*

Line 11-13:

In place of “Drug susceptibility tests for streptomycin, isoniazid, rifampicin, ethambutol, moxifloxacin and gatifloxacin will be done on positive cultures” amended to read as “Drug susceptibility tests for isoniazid, rifampicin, ethambutol and ofloxacin will be done on positive cultures. If the culture is resistant to isoniazid, rifampicin and ofloxacin, then drug susceptibility tests will be done for streptomycin, gatifloxacin and moxifloxacin”.

Page 9: Clinical assessments and Investigations:

*During treatment:*

*Line 1 and 2:*

In place of “susceptibility tests for isoniazid, rifampicin, ethambutol, moxifloxacin and gatifloxacin will be carried out on positive cultures”, amended to read as “drug susceptibility tests for isoniazid, rifampicin, ethambutol and ofloxacin will be carried out on positive cultures. If the culture is resistant to isoniazid, rifampicin and ofloxacin, then drug susceptibility tests will be done for streptomycin, gatifloxacin and moxifloxacin”.

Page 9: Clinical assessments and Investigations:

*During follow-up*

*Lines 7-9:*

In place of “drug susceptibility tests for isoniazid, rifampicin, ethambutol, moxifloxacin and gatifloxacin will be carried out on positive cultures”, amended to read as “drug susceptibility tests for isoniazid, rifampicin, ethambutol and ofloxacin will be carried out on positive cultures. If the culture is resistant to isoniazid, rifampicin and ofloxacin, then drug susceptibility tests will be done for streptomycin, gatifloxacin and moxifloxacin”.

Page 9: Clinical assessments and Investigations: During treatment:

Line 12:

Add “In addition, blood urea and serum creatinine will be done at 1st and 3rd monthly examinations also”. This has been recommended by the Data Safety Monitoring Board.

**15^th^ November 2006**

Amendment to the Protocol

**A study of efficacy and tolerability of Moxifloxacin and Gatifloxacin containing regimens in the treatment of patients with sputum-positive pulmonary tuberculosis, dated**

**27^th^ December 2003**

Following review of the interim findings of the above study, and recommendations by the DSMB on 14^th^ October 2006, and by the Ethics Committee Meeting of 4^th^ November 2006, and the decision taken by the Clinic Doctors Meeting of 13^th^ November 2006, the above protocol is being amended as follows:

Page 5, Regimen 1:

For the patients admitted to 2MHRZ3/2MHR3 regimen, moxifloxacin will be terminated from 15.11.06 and the treatment will be modified as follows:

In the Intensive Phase, patients will receive EHRZ3 instead of MHRZ3.

In the continuation phase patients will receive HR3 instead of MHR3.

At the end of 4 months HR3 will be extended for 2 more months.
